# HIV knowledge, disclosure and sexual risk among pregnant women and their partners in rural South Africa

**DOI:** 10.1080/17290376.2013.870696

**Published:** 2014-01-03

**Authors:** Molatelo Elisa Shikwane, Olga M. Villar-Loubet, Stephen M. Weiss, Karl Peltzer, Deborah L. Jones

**Affiliations:** a MA, Researcher at the Ekurhuleni Metropolitan Municipality, Department of Health and Social Development, South Africa; b PsyD, Research Assistant Professor, Department of Psychiatry & Behavioral Sciences, University of Miami Miller School of Medicine, Miami, FL, USA; c PhD, MPH, Professor of Psychiatry and Behavioral Sciences, University of Miami School of Medicine, Miami, FL, USA; d PhD, Dr Habil, Distinguished Research Fellow, Research Programme HIV/AIDS, STI and TB (HAST), Human Sciences Research Council, South Africa; e Extraordinary Professor, Department of Psychology, Free State University, South Africa; f Visiting Professor, ASEAN Institute for Health Development, Mahidol University, Thailand; g PhD, Research Professor, Department of Psychiatry & Behavioral Sciences, University of Miami Miller School of Medicine, Miami, FL, USA

**Keywords:** HIV, PMTCT, disclosure, partners, risk reduction intervention, Mpumalanga, VIH, SPTME, divulgation, partenaires, intervention de diminution des risques, Mpumalanga

## Abstract

Partner involvement has been deemed fundamental for the prevention of mother-to-child transmission (PMTCT) of HIV, although it remains difficult to achieve. This study aimed to explore the attitudes and behaviours of pregnant women and their partners who participated in a behavioural risk reduction intervention in six community health centres in the Mpumalanga province of South Africa. Qualitative methods only were used in this study. Women and their partners took part in four gender-concordant groups that addressed HIV, PMTCT, disclosure of HIV status and safer sex practices. The results indicate that men value and understand the importance of being involved in women's reproductive health, although some components of the PMTCT programme such as condom use were still met with some resistance. Participants demonstrated high levels of HIV- and sexually transmitted infection-related knowledge. Men lacked knowledge about PMTCT but were interested in acquiring information so that they could support their partners. All groups highlighted the emotional and physical benefits of disclosing one's HIV status. The involvement of men in antenatal care has the potential to prevent women from becoming infected with HIV both during pregnancy and post-partum when they are more vulnerable to infection and have a high risk of transmission to the infant. There is a need for interventions that focus on both increasing male involvement and promoting condom use during pregnancy.

## Abstract (FR)

La participation du partenaire est considérée comme fondamentale aux services de prévention de la transmission du VIH de la mère à l'enfant (SPTME), mais elle est difficile à obtenir encore aujourd'hui. Cette étude vise à explorer les attitudes et les comportements des femmes enceintes et de leur partenaire qui ont participé à une intervention de diminution des risques comportementaux réalisée dans six centres de santé communautaire dans la province du Mpumalanga, en Afrique du Sud. Des méthodes qualitatives ont été utilisées au cours de cette étude. Des femmes et leur partenaire ont formé quatre groupes avec concordance des genres. Ces groupes ont discuté du VIH, des SPTME, de la divulgation de leur état sérologique VIH, et des pratiques sexuelles à moindre risque. Selon les résultats, la participation à la santé génésique des femmes était très acceptée et appréciée par les hommes, et ce, même si certains aspects des SPTME, comme l'utilisation du condom, faisaient l'objet d'une certaine résistance. Les participants des groupes ont fait preuve de beaucoup de connaissances en matière de VIH et d'infection transmissible sexuellement (ITS). Les hommes manquaient de connaissance à propos des SPTME et souhaitaient obtenir de plus amples renseignements à ce sujet afin de pouvoir aider leur partenaire. Tous les groupes ont souligné les avantages émotionnels et physiques de la divulgation de l'état sérologique VIH. La participation des hommes aux soins prénataux peut éviter l'infection des femmes par le VIH pendant et après la grossesse, périodes durant lesquelles elles sont très vulnérables aux infections et que le risque de transmission au nourrisson est élevé. Il est nécessaire de réaliser des interventions axées sur l'augmentation de la participation des hommes et la promotion de l'utilisation du condom pendant la grossesse.

## Background

The HIV pandemic continues to threaten the lives of people in South Africa. It is estimated that approximately 5.6 million people in South Africa were living with HIV and AIDS in 2009, more than in any other country (UNGASS 2010). South Africa has the highest number of women infected with HIV/AIDS in the world, and women in sub-Saharan Africa account for nearly 70% of new HIV infections, with the primary mode of transmission being unprotected sex (UNAIDS 2011).

HIV incidence during late pregnancy is four times greater than in the non-pregnant population (Moodley, Esterhuizen, Pather, Chetty, & Ngaleka 2009). Exacerbating the problem further, women in South Africa may seroconvert during pregnancy following receipt of a HIV-negative test result (Kinuthia, Kiarie, Farquhar, Richardson, Nduati, Mbori-Ngacha, *et al.* 2010). Following an HIV diagnosis, some partners may not disclose their serostatus (Simbayi & Kalichman 2007) or take steps to protect the uninfected partner (Kalichman, Rompa & Cage 2005). In addition, the continuation of unprotected sex during pregnancy increases the risk of HIV-1 transmission (Mugo, Heffron, Donnell, Wald, Were, Rees, *et al.* 2011), supporting the urgent need to incorporate strategies in conjunction with prevention of mother-to-child transmission (PMTCT) education to prevent HIV transmission to women during pregnancy (Chen, Young, Brown, Chasela, Fiscus, Hoffman, *et al.* 2010).

Although condom use is known to prevent the sexual transmission of HIV, several studies have shown that the use of condoms is low among pregnant women. Onoya, Reddy, Sifunda, Lang, Wingood, van den Borne, *et al.* (2010) compared sexual behaviour among pregnant and non-pregnant women and discovered that while pregnant women had fewer male partners in the previous six months, they also reported less condom use and were less likely to request that their partners use condoms. Similarly, in Rakkai, Uganda, condom use was determined to be less prevalent during pregnancy (Gray, Li, Kigozi, Serwadda, Brahmbhatt, Wabwire-Mangen, *et al.* 2005). Manenti, Galato, Silveira, Oenning, Targino de Azevedo Simoes, Moreira, *et al.* (2011) found that the type of partner played a role as far as condom use was concerned. For example, if the woman was in a steady relationship, the prevalence of condom use was very low. In fact, among couples that associate condom use with contraception rather than risk reduction, sex may be more likely to be unprotected throughout pregnancy, greatly increasing the risk of HIV transmission.

Social norms and gender inequalities are some of the contextual factors that influence female behaviour and make them more vulnerable to HIV infection (Langen 2005). Gender-based power dynamics decrease a woman's ability to protect herself from HIV infection (Dunkle, Jewkes, Brown, Gray, McIntryre & Harlow 2004). Negotiating safer sex involves a woman's ability to comfortably discuss sexual matters, clearly assert her sexual needs and desires and avoid or refuse intercourse when her partner refuses to use a condom. This can often pose a challenge to women due to traditional gender role constraints (Melendez, Hoffman, Exner, Leu & Ehrhardt 2003).

For the past decade, male involvement has been recognised as an HIV programme priority area for the PMTCT of HIV (WHO 2007). Male partners play an important role in women's risk of acquiring HIV (Msuya, Mbizvo, Hussain, Uriyo, Sam & Stray-Pedersen 2006), particularly in terms of women's utilisation of the PMTCT programme. Key goals of the programme include increasing HIV testing rates among mothers and condom use among couples (Desgrees-du-Lou, Brou, Traore, Djohan, Becquet & Leroy 2009). Desgrees-du-Lou and Orne-Gliemann (2008) indicated that men's involvement in HIV counselling and testing could be understood in the context of understanding the couple's relationship, attitudes and communication patterns in regard to HIV and sexual and reproductive health.

Sexually transmitted infections (STIs) are a significant public health problem, particularly in developing countries (Mullick, Watson-Jones, Beksinka & Mabey 2005). STIs are highly prevalent among pregnant women in Africa and cause significant maternal and perinatal morbidity (Msuya, Uriyo, Hussain, Mbizvo, Jeansson, Sam, *et al.* 2010). Beyond posing a significant public health problem, STIs increase the susceptibility to and transmission of HIV (Malta, Bastos, Strathdee, Cunnigham, Pilotto & Kerrigan 2007). Knowledge of STIs and the ability to recognise their symptoms are therefore important because of their potential to accelerate care-seeking when needed.

Research on HIV knowledge in developing countries and the USA has revealed an inconsistent relationship between knowledge and HIV risk behaviour; some studies have even found a negative relationship between knowledge and sexual risk (Mbizvo, Msuya, Hussain, Chirenje & Stray-Pederson 2003; Peltzer 2003). Kershaw, Small, Joseph, Theodore, Bateau and Frederic (2006) maintain that HIV knowledge is still an important factor in promoting safer sex behaviours, making HIV education one of the key components of PMTCT interventions.

The study aimed to evaluate the impact of the combined ‘Partner-Plus’ intervention compared to the standard of care of male participation in the PMTCT process, which includes HIV testing by men, PMTCT protocol adherence and the use of sexual barrier products to reduce HIV transmission to mothers and their babies during pregnancy. This paper focuses on the intervention component and presents qualitative data from intervention sessions that explore the participants’ feelings, knowledge and attitudes towards HIV/AIDS, STIs, use of sexual barriers (e.g. male and female condoms), sexual negotiation and PMTCT.

## Aims and objectives of the study

The main aim of the study is to evaluate the impact of the combined ‘PartnerPlus’ intervention compared to the current standard of care of male participation in the PMTCT process. The objectives include

- to assess the impact of the intervention on men's HIV testing uptake;
- to measure PMTCT protocol adherence by HIV-positive pregnant women;
- to determine the impact of the intervention on the participants' use of sexual barrier products;
- to evaluate the impact of the intervention on mother-to-child transmission of HIV during pregnancy

## Methodology

The ‘PartnerPlus’ study recruited 239 couples from 12 community health centres in the Gert Sibande (4) and Nkangala (8) districts of the Mpumalanga province of South Africa. HIV prevalence in the antenatal clinics in the participating communities ranged from 15.4–36.8%. Community Health Centre Clinics in this randomised controlled trial were randomised into six control and six intervention sites. Participants were pregnant women who had completed HIV counselling and testing, were between weeks 24 and 30 of pregnancy and attended intervention group sessions with their partners. Couples were screened to ensure their status as primary sexual partners, and all participants provided informed consent and completed a baseline assessment. Participants at intervention sites received the standard of care (PMTCT) and participated in four ‘PartnerPlus’ sessions; those at the control sites received the standard of care (PMTCT) and attended four time-matched sessions (health-related videos on diabetes, hypertension, alcohol use and exercise). The data presented in this study are drawn from male and female intervention group sessions 1 and 2, which addressed HIV, STIs, safer sex, sexual negotiation and PMTCT.

## Intervention

The development of the ‘PartnerPlus’ intervention was guided by theories of reasoned action (i.e. intentions, influence, attitudes and subjective norms that influence beliefs about behaviour; Azjen & Fishbein 1980) and planned behaviour (i.e. perceived behavioural control influences intentions and behaviour; Schifter & Ajzen 1985) as predictors of sexual barrier use (Albarracín, Johnson, Fishbein & Muellerleile 2001). The intervention used a gender-concordant group format limited to 10 participants per group that has been described in previous literature (Jones, Chitalu, Ndubani, Mumbi, Weiss, Villar-Loubet, *et al.* 2009). Sessions were conducted for two hours a week for four weeks and were led by two trained counsellors. All sessions emphasised participation, experimentation and feedback, utilising role playing and the sharing of experiences to foster group cohesion. Issues addressed included STIs, HIV and PMTCT, safer sex and sexual negotiation; participants were provided with male and female condoms at the close of each session. Participants were not asked to disclose their HIV status, although some, especially the women, were open about their HIV status.

## Data analysis

The quantitative descriptive statistics were calculated using SPSS version 19. Intervention sessions were audio recorded, transcribed and translated from Sepedi, Ndebele and Zulu into English. Grounded theory was used to analyse the qualitative data. The theory is based on the process that helps the researcher systematically discover categories, themes and patterns that emerge from the data through coding and categorising the data into manageable units for analysis (Strauss & Corbin 1998). Open-ended questions led by trained counsellors were used to solicit thoughts and feelings on sexual health topics. Themes were identified by the first author through the extensive review process and were further reviewed by the second author until an 80% agreement was reached. Coding discrepancies were reconciled with the other authors through discussion and consensus.

## Results

Couples (*n* = 119) participating in the intervention had a mean age of 26 years (SD = 5.8) for women (range 18–41) and 30 years (SD = 7.1) formen (range 19–52). The majority of the participants had never been married, were unemployed and had educational levels ranging from grades 8–11. The data on the characteristics of the participants can be found in Table 1.

**Table 1.** Participant characteristics.

Variable	Women, *N* = 119	Men, *N* = 119
Mean age in years	26.1 (SD = 5.8) Range = 18–41 *N* (%)	30.5 (SD = 7.1) Range = 19–52 *N* (%)
Ethnicity		
Zulu	42 (35.3)	41 (34.5)
Ndebele	32 (26.9)	33 (27.7)
Sepedi	25 (21.0)	25 (21.0)
Other	20 (15.1)	20 (16.7)
Marital status		
Married	14 (11.8)	13 (10.9)
Never married	89 (74.8)	88 (73.9)
Living together	16 (13.4)	18 (15.1)
Educational level		
Grade 7 or less	7 (5.9)	8 (6.7)
Grade 8–11	68 (57.1)	52 (43.7)
Grade 12 or more	44 (37.0)	59 (49.6)
Employment status		
Employed	18 (15.1)	45 (37.8)
Unemployed	97 (81.5)	72 (60.5)
Student/leaner	4 (3.4)	2 (1.7)
Number of children		
No children	47 (39.5)	51 (42.9)
One	46 (38.7)	36 (30.3)
Two	14 (11.8)	18 (15.1)
Three	9 (7.6)	11 (9.2)
Four or more	3 (2.5)	3 (2.5)
HIV-positive status	37 (31.1)	13 (10.9)
Knowledge of partner HIV status	68 (57.1)	105 (88.2)
Session 1: Attendance		
Attended	94 (79.0)	91 (76.5)
Did not attend	25 (21.0)	28 (23.5)
Session 2: Attendance		
Attended	96 (80.7)	92 (77.3)
Did not attend	23 (19.3)	27 (22.7)

## Intervention themes

The results from the intervention sessions are presented according to the themes identified, which include knowledge of STIs, HIV/AIDS and PMTCT, stigma, disclosure, acceptability of the use of sexual barrier products and sexual negotiation. These themes were derived from major issues that were addressed during the intervention.

## Sexual barrier products and sexual negotiation

### Acceptability of condoms

Experiences with and feelings towards the use of sexual barriers (i.e. male and female condoms) was a salient theme throughout the session discussions. Group counsellors explored prior experiences with sexual barriers as well as reasons for non-use. Questions and concerns were raised, and misconceptions were dispelled within the group discussion. Demonstrations of sexual barrier products were conducted, and participants were provided with male and female condoms and were asked to provide feedback about their experiences in subsequent sessions.

Both male and female participants felt that the use of condoms was an important strategy to protect against infections. However, women did not feel that it was necessary for them to use condoms if they were pregnant:

I did not use condoms because I did not see the use since I am already pregnant. (Female, Siyathemba clinic)

I have not used condoms since I found out I was pregnant. (Female, Vlaklaagte clinic)

When asked whether participants were practising safe sex, one woman replied:

I do practice safe sex, or I can say I will after the child is born. (Female, Allemansdrift clinic)

One man shared his perspective:

I cannot use condoms when my partner is pregnant because the child must get my vitamins. (Male, Moloto clinic)

Both men and women were uncertain about the use of the female condom. One man said: ‘I prefer the male condom because the female condom seems too complicated to use.’ Concerns about the female condom were also discussed: ‘So these rubber bands will not cause the woman any pain?’ ‘Won't this female condom cause me any pain?’

Although some men and women showed interest in trying to use the female condom, most preferred the use of the male condom:

It does not look easy; I think the male condom is easy to use unlike the female condom – it looks very difficult to use. (Female, Allemansdrift clinic)

I prefer the male condom because it is easier to use and it is accessible. (Male, Siyabuswa)

### Sexual negotiation

The majority of the women maintained that they continued to find it difficult to negotiate safer sex practices with their partners, even following their male partner's participation in the intervention. This problem was more prevalent among women who stated that they were economically dependent on their partners. Some social and cultural norms also fuelled this challenge and made women more apprehensive to discuss sex with their partners. During the discussion of sexual negotiation, men and women were encouraged to roleplay problems they anticipated encountering when discussing safer sex practices with their partners. Scenarios were played out, and subjects discussed how to approach one's partner regarding safer sex practices. Questions posed to the group included, ‘Why should a pregnant woman use a condom?’, ‘Why should a couple committed to each other use a condom?’ and ‘How would the other partner react to the sudden introduction of a condom in the relationship?’

Women discussed their partner's reactions to using a condom, and it became clear that men did not like using condoms:

He did accept it (a condom), but it was really hard for him because he said he was not used to using condoms but he got used to the idea even though it was hard for me to see him unhappy because I wanted us to use protection. (Female, Moloto clinic)

Participants practised their negotiation skills during the intervention sessions and gave feedback about their experiences. It became clear that talking to partners about condom use seemed to be more of a challenge for women than men. When participants were asked by the counsellor or a fellow group member how they would broach the subject of condom use, one man said:

I would just tell her that we have to start using condoms because I went to get an HIV test performed, and I was told to use condoms until both of us are tested and we know our status. (Male, Vlaklaagte clinic)

One woman responded in this way:

That would be difficult, but I will just have to find a way of talking to him. (Female, Moloto)

Another woman said:

I would try by all means to make him understand the importance of using condoms and make him know that they are very helpful and that they will be protecting our unborn child from unwanted infections and diseases. (Female, Siyabuswa clinic)

Women provided candid feedback about negotiation with partners surrounding condom use. One woman's experience was not positive:

My partner is a very difficult man; he wanted nothing to do with what I had to say. (Female, Winniefred Maboa clinic)

Another woman gave advice to other group members on how to approach their male partners:

You have to tell him politely because some men don't want to listen to what we say when they want to have sex. (Female, Moloto clinic)

For some women, the condom negotiation experience was positive:

I told my partner we have to use condoms, and when he asked I told him that we had to protect our unborn child and he understood after I explained to him how our child would be protected and won't get infected. (Female, Allemansdrift clinic)

I spoke to my partner and he agreed to use condoms, but he said he wanted to use the male condom not the female condoms; we are now using protection, and I am pleased with our agreement. (Female, Moloto clinic)

Men were asked how they would feel if their partners wanted to start using condoms with them. One man said, ‘I personally would think she does not trust me.’ Another man said, ‘I would think she is sick or maybe she was cheating on me or she has cheated on me.’

### HIV and PMTCT knowledge, disclosure and stigma

#### HIV-related knowledge

Knowledge of modes of transmission were discussed in both the male and female group sessions. Participants expressed their fears about HIV, discussed myths associated with HIV/AIDS and explored new HIV-related knowledge. Participants demonstrated some knowledge of HIV, and women demonstrated that they had not only knowledge but also experience. Women openly discussed their reactions during the sessions:

HIV is actually a very terrible disease because I had a brother who was infected with the virus and he would not take medication in time and did not follow up on his check-ups and the virus took over his body and the disease took him. (Female, Siyathemba clinic)

I personally have the virus and only found out after being sick for a very long time; the doctors did all the tests they could and eventually I tested for HIV. (Female, Siyathemba clinic)

I know the virus personally because I tested positive back in 2009 and have been living with it ever since. (Female, Moloto clinic)

#### PMTCT knowledge

Because all of the women enrolled in the study were pregnant at the time of enrolment, PMTCT was the most salient theme throughout the group discussions. The facilitators and participants shared information about the modes of transmission from mother to child and prevention strategies. Participants, mostly men, were curious about the process by which PMTCT occurs. In addition, treatment and prevention issues were discussed in detail. The importance of testing for HIV during pregnancy was highlighted, and participants felt it was necessary for one to test for HIV if PMTCT was to be successful. Women demonstrated more familiarity with PMTCT than their male counterparts:

The child can get infected if the mother has unprotected sex while she is pregnant. (Female, Allemansdrift clinic)

The child can get the HIV virus during birth if the mother is HIV-positive because of the blood that is present when she gives birth. As we know, it happens that the child may get cuts during delivery, and the child blood can get mixed with that of the mother. (Female, Winniefred Maboa clinic)

Men had more questions to ask about PMTCT, indicating that they needed more information. Some of the questions asked included: ‘what kind of medication should the HIV-positive woman take? Is it possible for the baby to be HIV-negative?’ ‘Why should the child be exclusively breastfed for only six months, and what will happen thereafter?’

Some men were surprised by the information that was shared by other men during the sessions. One man said:

In our culture, we as men are not really supposed to know much about women when they give birth and what goes on when they are giving birth. (Male, Siyathemba clinic)

Another man said:

You are right, but unfortunately we are living in times where we have to take part in what takes place with our partners because we are living in a world where HIV is taking over our lives and we need to be very careful. (Male, Siyathemba clinic)

#### Disclosure of HIV status

Disclosure of HIV status was among the more salient topics discussed by both male and female groups. The quantitative data collected indicated that 44 of the participants were HIV seropositive, of whom only 20 had disclosed their status to their partners. In particular, participants focused on whether to disclose their status to their partners and determining the right time to disclose. Various reasons for or against disclosure were also explored by the participants during the discussion. Some participants felt that disclosure is important, especially when the disease progresses, because the person will then need help from others:

It is important because if you were to become sick or need help, the person you have told will know what they should do and how to help you. (Male, Allemansdrift)

Another participant highlighted the benefits of disclosure:

I think disclosure is important to a person because when you disclose your status it becomes easy for you to live your life, not having worries of what people would say and you will live a healthy life without stress. (Female, Winniefred Maboa)

Although all of the participants felt that it was important to disclose one's HIV status, they also believed that one should not feel pressure to disclose:

I think that it should be one's choice to disclose their status; it should not be by force. If the person feels they do not want to disclose then it is their choice. (Female, Vlaklaagte clinic)

I think the only people you should disclose to are the people you trust, believe will not tell anyone and will be by your side when you need support. (Female, Vlaklaagte clinic)

Disclosure to their male partners was an area that created anxiety for many women. The general feeling among the female participants was that if a woman disclosed her HIV-positive status to her partner, the man would leave her:

If you were to let your partner know about your status, then they will leave you. (Female, Siyathemba clinic)

I think it is the right thing to do because firstly it will mean that your partner will have to get tested as well, and in other cases, you will find that he already knew his status and was waiting for you to make the first move. (Female, Siyathemba clinic)

One woman had this to say about her disclosure experience:

When I had to first disclose my status, it was just after I had a baby who shortly died a few months after birth. At first it was not easy because I told my brother who I was close to and he did not take the matter very well; he treated me differently, but I understood where he was coming from. I later had to tell my mother who was very shocked but later accepted my status. I have been living openly with my status for years and have been on treatment without any complications. Me and my partner have been sexually involved without any troubles because we both know our statuses. (Female, Moloto clinic)

#### HIV-related stigma

Stigma associated with HIV was highlighted by both male and female groups when issues related to non-adherence to treatment were discussed. When asked why people living with HIV are not adhering to their treatment, the participants responded:

I think the stigma associated with the virus makes it hard for people to accept their status. (Male, Siyabuswa clinic)

I think it is because they somehow feel embarrassed to take ARV in front of everyone. (Female, Vlaklaagte clinic)

Several reasons were given as to why people do not adhere to their treatment, which included being embarrassed to go to the clinic to take medication, denial and stress that could lead people to forget to take their pills.

### Increased STI-related knowledge and experience

Both the men and women's groups showed considerable knowledge about STIs and were able to openly share their personal experiences with STIs, as the below statement indicates:

I had some kind of STI and it took me some time to recognise it; each time I went to bath I would find some kind of dirt on my underwear that had dried up, and it gave me a fright to see that but I continued having sex with the condition I had. After intercourse, I continued to have this hot sensation when I had to pass urine, and later I decided to go to the clinic to have it checked out. They told me it was indeed an STI, and they gave me Doxicycline and asked where my partner was; she was given the same treatment as well. (Male, Moloto Clinic)

Participants felt that treating STIs, particularly during pregnancy, was important because they can end up affecting the child. After being shown a poster with pictures of different STIs, participants felt that looking after their bodies and being aware of any changes to their private parts was important, especially to the female participants:

I think we also as women should check ourselves if you find something you do not know or if it was not there before, it is important that if you get such things to get the necessary treatment as soon as possible; do not let it grow bigger or get worse, and get to a stage where it is not treatable anymore. (Female, Vlaklaagte clinic)

Another female participant said:

It is also important that you do not agree to have sex in the dark because that is when they (men) will be hiding these things from you, so I think the best thing is for us not to switch off the lights. (Female, Vlaklaagte clinic)

## Discussion

The study explored the knowledge, feelings and attitudes towards HIV/AIDS, the use of sexual barrier products and sexual negotiation among pregnant women and their partners who participated in gender-concordant sexual risk reduction intervention groups. Participants in both groups were open about discussing STIs, HIV, PMTCT and HIV status disclosure.

Given the high level of HIV incidence during late pregnancy in South Africa (Moodley *et al.* 2009), it is important that pregnant women and their partners use condoms during sexual intercourse. Traditional gender roles in South Africa make it difficult for women to suggest condom use to their partners without undermining the trust in their relationship (Falnes, Moland, Tyllerskar, de Paoli, Msuya & Engebretsen 2011). Although men in the current study acknowledged that the use of condoms is important in preventing infections, they were not happy to be told by their female partners to use condoms and felt that their partners did not trust them or that their female partner was cheating if she suggested condom use. Among those that accepted the use of a condom, most preferred the male condom over the female condom. The apparent lack of safer sex practices during pregnancy in this population is especially disturbing given the high rates of transmission during pregnancy and an unwillingness to use condoms during this vulnerable period. It may be especially useful to increase men's awareness of their own heightened vulnerability to HIV infection during their female partner's pregnancy (Mugo *et al.* 2011).

Men rarely participate in ANC/PMTCT services mainly because these are traditionally and programmatically a women's domain (Orne-Gliemann, Tchendjou, Miric, Gadgil, Butsashvili, Eboko, *et al.* 2010). In addition, the institutional structures and women-centred PMTCT and ANC services make it difficult for men to participate. Men who participated in this study indicated that reproductive health issues were traditionally a women's responsibility, although they accepted that it is important for them to be involved. They had a significant amount of HIV knowledge but did not have much information about PMTCT.

The belief that disclosure of one's HIV status to sexual partners or significant others will lead to rejection and stigmatisation (Mlambo & Peltzer 2011) appears less important to the majority of participants in the current study. Although disclosure is seen as difficult and participants felt that they should not be pressured to disclose, they highlighted the health and emotional benefits of disclosure. This conclusion supports the findings of other studies that disclosure may alleviate the stressful burden of concealment and increase material and emotional support from others (Bouillon, Lert, Sitta, Schmaus, Spire & Dray-Spira 2007).

A lack of knowledge regarding STI transmission, detection and treatment and perceived STI-related stigma are both linked to delays in care-seeking among patients (Malta *et al.* 2007). The participants in this study appeared knowledgeable about STIs and did not express concerns with stigma; several shared with other group members that they had been previously diagnosed with an STI. Participants were candid in sharing their experiences with infection, demonstrated knowledge surrounding the identification of STIs and stressed the importance of seeking treatment during pregnancy.

This study sought to explore attitudes and behaviours among couples during pregnancy regarding HIV, PMTCT and safer sex practices. The results highlight the urgent need for clinical care teams to encourage protected sex during pregnancy and emphasise male involvement to enhance condom uptake. Future clinical interventions should focus on increasing male awareness and motivation to use condoms during this period in which mother, baby and father are all more vulnerable to infection.

## Conclusion and recommendations

Condom use neglected during pregnancy increases the risk of HIV transmission. Factors that contribute to the non-use of condoms include women's inability to negotiate condom use with their partners, economic dependency on their male partners and fear of partner violence. The findings of this study also show that male partners do not have knowledge of PMTCT and view it as a woman's responsibility. Therefore, there is a need to implement long-term sexual risk reduction interventions that target men as the primary decision-makers in relationships. HIV counsellors and nurses should also address the issue of HIV transmission during pregnancy to reduce the risk of seroconversion in this population.
